# Combined Use of Biostimulation and Deficit Irrigation Improved the Fruit Quality in Table Grape

**DOI:** 10.3390/plants14030485

**Published:** 2025-02-06

**Authors:** Susana Zapata-García, Pablo Berríos, Abdelmalek Temnani, Pedro J. Espinosa, Claudia Monllor, Alejandro Pérez-Pastor

**Affiliations:** 1 Departamento de Ingeniería Agronómica, Universidad Politécnica de Cartagena (UPCT), Paseo Alfonso XIII, 48, 30203 Cartagena, Spain; susana.zapata@upct.es (S.Z.-G.); pablo.berrios@upct.es (P.B.); abdelmalek.temnani@upct.es (A.T.); 2 Europe, Middle East & Africa Region (EMEA) Plant Health Portfolio, FMC Agricultural Solutions, 28046 Madrid, Spain; pedro.espinosa@fmc.com; 3 Plant Health Portfolio, FMC Agricultural Solutions, 28046 Madrid, Spain; claudia.monllor@fmc.com

**Keywords:** berry color, biostimulant, precision irrigation, sustainability, yield precocity

## Abstract

This study aims to determine the effects of four different biostimulation treatments—composed of microorganisms, seaweed, and plant extracts—on the yield and quality traits of table grapes. Those treatments are compared with an untreated control treatment and tested under two different irrigation schedules: (i) Farmer Irrigation (FI), according to farmer criteria, and (ii) a deficit irrigation program, Precision Irrigation (PI), irrigated as FI, except during the post-veraison period when a 10% soil water depletion was allowed to mitigate the lixiviation. The water inputs in the treatments under PI were reduced by 30% without affecting the total yield but still promoting harvest precocity—an effect that was enhanced by the biostimulated treatments. This deficit irrigation program also stimulated berry growth and a higher maturity index. The different biostimulation treatments led to an improvement in the physical and chemical quality traits of the grapes; under FI, they showed a bigger size and a greater weight than the non-biostimulated treatment, while under PI, they showed a higher soluble sugar concentration and maturity index. Regardless of the irrigation program, the commercial berry color proportion was increased in all the biostimulated treatments, reducing the percentage of green berries. The combined use of biostimulation and PI can promote more efficient and sustainable farming practices, promoting fruit yield precocity and quality of the grapevine in drought-prone regions.

## 1. Introduction

Table grapes are a significant horticultural crop worldwide, valued for their fresh consumption due to their sensory and nutritional attributes. Globally, grapes are one of the most valuable fruit crops, with a farm gate value of approximately USD 68 billion [1], including fresh consumption products, raisins, grape juice, and wine.

Vines are usually cultivated in temperate areas, with the largest cultivated area for vineyards in the world being found in Spain and accounting for 13.8% of the global vineyard area in 2021 [2]. The Murcia Region together with the Valencian Community are the two key provinces for table grape cultivation, accounting for 63% and 33% of Spain’s table grape production, respectively [3]. Additionally, 68.4% of this combined region’s production was exported in 2021 [3,4]. In order to maintain the export levels, the country has been focusing on varietal reconversion and entering into new markets [5]. One of these varieties is ‘Sweet celebration’, a seedless variety, with bright dark red berries, a crunchy texture, and a sweet taste [6].

To ensure the adaptation of those varieties, farmers must adopt different measures, such as pruning, irrigation and others soil practice managements [7].

However, abiotic stressors, including primarily drought, extreme temperatures, and salinity, are responsible for 60 to 70% of the yield lost [8]. In drought-prone regions like the Mediterranean basin, farmers have been using different techniques, such as regulated deficit irrigation (RDI), to deal with water scarcity while also trying to increase water productivity [9]. In table grapes, researchers have reported that during the post-veraison period, maintaining the stem water potential above −1.2 MPa does not affect the yield [10,11], as this period is categorized as a non-critical one [12]. Although, taking this threshold into consideration for pre- and post-veraison periods could have a negative effect on yield [13].

In addition, RDI strategies have been shown to be particularly useful in the management of this crop, as they can mitigate the excessive vigorous growth of the vines [14,15]. RDI strategies have been shown to enhance different berry quality traits, such as an increase in total soluble solids or the coloration of the berries [16] and an accumulation of bioactive compounds in the skin, including anthocyanins or resveratrol [17]. While other quality traits as firmness [10], cold storage, and shelf life remain unaffected [18].

During the last decades, diverse techniques have been widely used in viniculture, such as the application of plant growth regulators (PGR) and, lately, biostimulants—substances or microorganisms that, when applied to plants, enhance their nutrient use efficiency, abiotic stress tolerance, or crop quality traits by mechanisms that do not directly add fertility to the soil [19]. In this regard, the foliar application of brasinosteroids at the onset of veraison was shown to enhance the color, anthocyanin pigmentation, and total soluble solids content in berries [20]. Similar findings in terms of berry quality have been found when abscisic acid was sprayed at 7 and 21 days after veraison, increasing anthocyanin accumulation in the berry skin, which then affected the expression of biosynthetic genes and transcription factors involved in coloration, promoting flavonoid and anthocyanin synthesis [21]. Moreover, these substances can increase the protection of the crop against certain diseases, such as Pierce’s disease [22].

Biostimulants were initially studied as substances that mimic the effects of PGR due to their phytohormone content [23]. Indeed, some plant extract biostimulants promote the same pathways as abscisic acid in order to enhance berry coloration [24] however, this trend has been attributed to only one of the extract components—oxylipins [25]. The effect of biostimulants and their interaction with PGR on the early ripening of grapes has been studied, finding an optimum biostimulant dose but concluding that it is independent of the PGR used (gibberellic acid, GA_3_) [26]. Biostimulants do not only affect the color of grapes, they have also been reported to have an influence on other quality traits, such as the organoleptic profile. As reported by Kok and Bal [27], after 3 foliar applications of seaweed extract and humic acids, both treatments increased the volatile terpenes concentration in berries. In addition to improving berry quality traits, biostimulants have been used to mitigate biotic and abiotic stress in viticulture, as it has been recently reviewed by Monteiro et al. [28], who highlight the potential of biostimulants as an alternative to reducing the chemical inputs in agriculture.

The combination of both strategies, RDI and biostimulation, has been studied by Zapata-García et al. [29] as a tool to increase water productivity in drought-prone regions. They found that the soil and plant water status indicators were affected by RDI, while some physiological parameters, such as root growth and its colonization by mycorrhizae, were promoted by the different biostimulants. Researchers highlight the sustainability and economic viability of this technique for viticulture, contributing to sustainable development goals [30].

However, grape quality traits are key parameters for market acceptance. Therefore, the objective of this research was to assess the effects on the yield precocity and quality of table grapes biostimulated during two consecutive years and in combination with deficit irrigation in post-veraison during the second year.

## 2. Results

### 2.1. Soil and Plant Water Status

The weekly farmer irrigation (FI) schedule ranged from 50 or 20 m^3^ ha^−1^ in May 2021 or 2022, increasing gradually according to the climatic demand. Between July and August, the months of highest demand, the weekly irrigation volume reached its peak with 350 m^3^ ha^−1^. Thus, at the end of the season, the total irrigation volume under the farmer irrigation (FI) program amounted to 4411 m^3^ ha^−1^ and 4403 m^3^ ha^−1^ in 2021 and 2022, respectively (Table 1).

The deficit irrigation program carried out in 2022, precision irrigation (PI), was managed like FI until the 49 DAFB, 4 days before the 50% veraison (1000 °C GDD). By this time, when FI increased the water inputs, averaging 250 m^3^ ha^−1^ per week, the daily irrigation volume amount applied under PI was reduced. As a result, by the end of the 2022 season, the PI program ended up with an irrigation water amount of 3067 m^3^ ha^−1^, saving 1336 m^3^ ha^−1^ compared with the FI program that year (Table 1).

Before veraison, the water content in the soil profile between 20 and 40 cm depth (θ_20–40cm_) increased by 9.90%. Because the irrigation was reduced under the PI program from the post-veraison period until the end of harvest, the θ_20–40cm_ in this stage did not vary, as the water applied equaled the water demand. During this same period, the θ_20–40cm_ under the FI program was increased by 2.06%. As a consequence, the stem water potential (Ψ_s_) during this same period was significantly reduced under the PI program compared with FI by an average of −0.20 MPa (Table 1).

### 2.2. Yield

**Figure 1:**
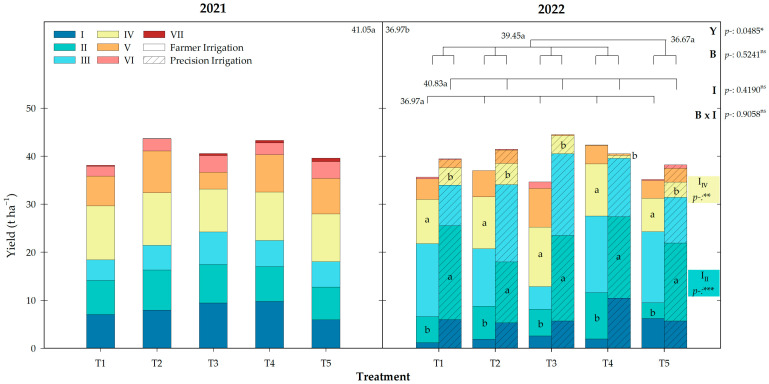
Total yield for biostimulation treatments under different irrigation programs (farmer and precision irrigation) during the years 2021 and 2022. The stacked bars represent the different harvests in each treatment; different lowercase letters inside them indicate significant differences between irrigation programs on the same harvest day according to Duncan’s test (*p* < 0.05). The average value for each factor (Y: year; B: biostimulation; I: Irrigation) is shown together with the statistical differences according to Duncan’s test (*p* < 0.05). Colored boxes indicate the *p*-value significance for the irrigation factor on those harvests. *: *p* < 0.05; **: *p* < 0.01; ***: *p* < 0.001; ns: not significant.

The fruit yield for the different harvests carried out in 2021 and 2022 are represented in Figure 1. In 2021, the untreated treatment, T5, totaled 39.6 t ha^−1^, while the biostimulated treatment non-significantly increased the harvest by 1.78 t ha^−1^. Among them, the T2 and T4 treatments stood out, corresponding to *Ascophyllum nodosum* (AN) extracts alone or in combination with microorganisms, respectively.

During 2022, the yield under the FI program was reduced by 4 t ha^−1^. Despite this, the maximum yields were achieved by the same treatments as the previous year, with T4 being 20% higher than the non-biostimulated treatment, T5.

The PI program carried out in 2022 increased the harvest obtained that year to 3.86 t ha^−1^. The treatment T3, in which a *Bacillus*-based biostimulant was applied, showed the highest yield, increasing the harvest obtained in T5 by 17%.

No significant differences were shown between the FI and PI irrigation programs, but earliness in the harvest was observed, being significantly different at the harvests II (*p*-value < 0.001) and IV (*p*-value < 0.01) (Figure 1). This harvest precocity was linked to the harvested bunch numbers, being 10.79 and 40.94 in the second harvest, while the fourth harvest accounted for 21.81 and 8.15 bunches under FI and PI, respectively.

The precocity promotion caused by PI affected the treatments differently. The average yield increase in the early harvests (I to III) with respect to the FI program was 16.3 t ha^−1^ for the biostimulated vines, whereas T5 increased to only 7.2 t ha^−1^. As a result of this combination, the biostimulated treatments under PI (B_PI_) got 89% of the total harvest in those cuts, while the non-treated control under farmer irrigation (T5_FI_) got 69%.

### 2.3. Berry Quality

#### 2.3.1. Physical Traits

During the pre-conditioning year, 2021, no effect was observed in the physical quality traits of grapes. However, in the second season, the average berry size and weight were reduced under the FI program, obtaining grapes that were 2 mm and 1 g smaller than 2021, while the average hardness was increased by 8 points. Under the FI program, the biostimulation treatments showed significant differences in all the physical traits analyzed. Particularly for that condition and year, the untreated control, T5, had the smallest berries in size and weight, while all the biostimulated treatments increased those parameters by an average of 29 and 21%, respectively. The T2 treatment had the berries with the highest diameter, while the hardness, measured through the shore A scale, was higher in T3’s berries compared with the rest of the treatments.

During the 2022 season, the effects of the irrigation factor on physical quality varied between the biostimulated and non-biostimulated grapes. There was a significant effect on the non-biostimulated grapes (T5_FI_), which averaged 16.86 mm and 3.97 g, while in contrast, the biostimulated ones under the same irrigation program (T1–T4, B_FI_) did keep the same size and weight of their berries (Table 2). The average size slightly decreased under the PI program, whereas the berry weight and hardness were not affected.

**Figure 2:**
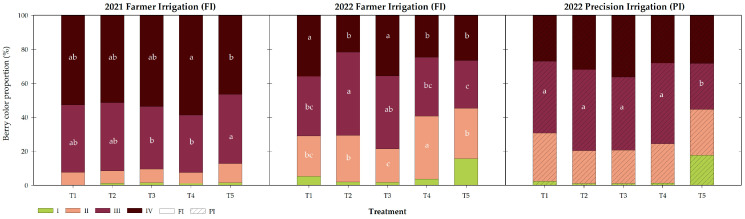
Berry color proportion (*n* = 200) for the different treatments at the first harvest of each season and irrigation program. Different letters indicate significant differences for the same color category between the treatments within the same irrigation program and season according to Duncan’ tests (*p* < 0.05).

The color proportion of the first harvested berries for each season, irrigation program, and biostimulation treatment is represented in Figure 2. For both seasons, the green berries proportion, classified as Category I, is the minority, representing around 1% under the FI program during 2021. It increased in 2022 season under both irrigation programs, particularly for treatment T5 (T5_FI_ 16%, T5_PI_ 18%). The biostimulated treatments (T1–T4) under FI also showed an increase in the percentage of Category I berries to 3.2%, while it remained at 1.4% under the PI program.

Color Category II corresponds to a ripening stage, but not the full veraison color. In 2021, it represented around 8%, without differences between the biostimulation treatments. It increased in 2022, under FI and PI, representing 28 and 24% of the total berries, respectively. This category showed differences between the biostimulation treatments under FI, with T4 and T3 being the ones with the highest and lowest number of berries in this category, respectively.

Categories III and IV contain the accepted berry coloration by consumers, covering 91% of the harvested berries in 2021. Although their summatory did not differ between treatments, it did within the individual categories, with T5 and T4 being the ones with the highest number of berries in Categories III and IV, respectively. The percentage of berries in commercial categories decreased in 2022 under FI, totaling 55% for T5 and 70% for biostimulated treatments. Among the biostimulated treatments, T3 and T4 respectively showed the highest and lowest proportion of berries in the consumer-accepted categories, being 78 and 59%, respectively.

When biostimulation was combined with deficit irrigation in the post-harvest stage, more homogeneity in the color was found. Under the PI program, Category IV was similar for all the treatments, averaging 31% of the berries. Category III did change, being reduced in T5, involving 27% of the total, while the proportion of the biostimulated treatments in this category averaged 45%.

Assessing the year factor under the FI program, for the non-biostimulated treatment, T5, all categories changed their proportion between 2021 and 2022 (*p* < 0.0001). For the biostimulated treatments, the effect on Category III was not observed (*p* = 0.84), showing a similar proportion of berries in this category as in the previous year.

The irrigation program in 2022 also proved to have an influence on Categories I (*p* = 0.008) and II (*p* = 0.018) for the biostimulated treatments, while it did not influence any category percentage for the untreated treatment (T5).

#### 2.3.2. Chemical Quality

The total soluble solid (TSS) and the titratable acidity (TA) were not significantly influenced by the biostimulated treatments under farmer irrigation during 2021 or 2022. Although, there were seasonal differences in the chemical quality traits, as the average values in 2021 were 18.45 °Brix and 0.57 mg L^−1^ and 16.74 °Brix and 0.38 mg L^−1^ in 2022 (Table 3). Due to the decrease in TA, the maturity index (MI) was also increased in 2022.

The irrigation reduction carried out in 2022 significantly influenced the chemical quality of the grapes, increasing the TSS and reducing the AT, resulting in an increase in the MI under the irrigation reduction program. The biostimulated treatments under the PI program punctually showed significant differences in TA, decreasing in T3. The TSS increased in all the biostimulated treatments (T1–T4, B_PI_), with respect to the untreated treatment, T5. The ratio between these parameters, MI, was also affected by the combination of PI and biostimulation, obtaining an average index of 47 for B_PI_, 3 units higher than T5_PI_.

## 3. Discussion

Reducing the irrigation water volume by 30% during the post-veraison period resulted in a decrease in soil water storage under the precision irrigation (PI) program compared with the farmer irrigation (FI) program. This water stress also led the stem water potential (Ψ_s_) to be reduced. Vines under the PI program reached Ψ_s_ values of −0.86 MPa, while those under the FI program remained at −0.66 MPa (Table 1). The lower values reached for Ψ_s_ under the PI program were below the threshold established by other authors [10,11]. Particularly, Acevedo-Opazo et al. [11] pointed out that the threshold value of –1.2 MPa can be used to optimize the soil water availability, irrigation scheduling, yield, and grape quality.

The yield was not compromised under the PI program carried out in 2022. On the contrary, this water restriction caused an increase in yield if it is compared to the FI program, raising it by 3.86 t ha^−1^, equaling the total 41 t ha^−1^ obtained the previous year. The decrease in the FI yield from 2021 to 2022 can be attributed to the alternate bearing behavior commonly observed not only in fruit trees but also in vines [31]. Our data suggest that reducing the irrigation during a non-critical period not only improves the irrigation water productivity but can also mitigate alternate bearing.

Compared with the untreated treatment, T5, the biostimulation treatments under the FI program non-significantly increased the yield by 1.78 and 2.28 t ha^−1^ in 2021 and 2022, respectively. Particularly, the treatments that most improved the yield under FI were T2 and T4, both composed of *Ascophyllum nodosum* (AN) extract, alone or in combination with microorganisms. In contrast, T4 was the only treatment that numerically decreased its production under PI, while T3, composed of microorganisms, was the treatment that most enhanced the yield, reaching 44.51 t ha^−1^.

The deficit irrigation programs also promoted the harvest precocity, as it has been pointed out by other researchers [12]. All treatments under PI showed significant differences with respect to FI in harvest II and IV, attributed to the number of bunches harvested. The earliness of the harvest promoted by the irrigation program was particularly notable in the biostimulated treatments (B_PI_), accounting for 89% of the total harvest in the first three cuts. This resulted in an average yield increase of 16.3 t ha^−1^ with respect to the same treatments under FI, while the non-treated control only showed an increase of 7.2 t ha^−1^, highlighting the synergistic effect of biostimulation and deficit irrigation.

In a similar way as it occurs with the yield, berry growth in 2021 was not affected by the biostimulation treatments. In fact, between the years 2021 and 2022, berry growth diminished, the size and weight of the first harvest were significantly reduced, and the hardness was increased (Table 2). Berry growth can be affected by a water deficit, depending on the level and the period during which it is applied. Pinillos et al. [12] found that berries have rarely grown smaller after a post-veraison irrigation reduction of 25 and 50%. In 2022, the non-biostimulated grapes irrigated under FI were smaller than the biostimulated ones—T5_FI_: 3.97 vs. B_FI_: 5.12. However, this trend was not observed under the PI program—B_PI_: 4.96 vs. T5_PI_: 4.70 (Table 2). The application of the different biostimulants tested in this trial was shown to improve the physical quality of the berries, mainly under farmer irrigation in 2022, when the berry weight and size increase. This was likewise observed by Ferrara and Brunetti [32] and Irani et al. [33] after biostimulating grapevines with humic acids. The reason this effect was not observed under the PI program is the same as for the trials carried out by Gutierrez-Gamboa et al. [34], where it was shown that the effect of the same biostimulant can be modified depending on the climatic variables, such as the seasons and rainfalls, affecting for example to the berry size between. 

The chemical quality traits, titratable acidity, and total soluble solids (TSS) also decreased in 2022 compared with 2021 (Table 3). However, these parameters were not influenced by the biostimulation treatments during any of the years under the farmer irrigation program. The deficit irrigation program that was applied in 2022 influenced the quality traits of the grapes, decreasing the TSS, as is typically reported for table grapes under deficit irrigation conditions [16,33]. Under the precision irrigation program, even though the different treatments showed differences in the TSS levels, all the biostimulated treatments increased the TSS levels compared with the untreated control (T5). This is similar to what has been observed after biostimulation with seaweed extracts in table grapes [33], although some plant extracts were able to decrease TSS levels while maintaining the same levels of titratable acidity (TA) [35]. The TA decreased in T3 under PI. This treatment was composed of microorganisms and, in this way, its maturity index (MI) increased like the rest of the biostimulated treatments under PI, which increased the MI by an average of 3 points with respect to the non-biostimulated treatment under the same irrigation program.

In 2021, the average percentage of berries in Categories III and IV for biostimulated treatments and untreated control represented the vast majority of the harvested berries, being 91.7 and 87.3%, respectively. Berry coloration decreased significantly during the 2022 season, but biostimulation played a fundamental role in enhancing it, representing an average of 69.9% (B_FI_) and 54.7% (T5_FI_) of the berry color proportion for Categories III and IV. Particularly, the T1 (plant and seaweed extracts) and T3 (microorganism) treatments increased the berry color proportion in Category IV, while the T2 (AN extract) and T3 treatments in Category III were the same as the untreated control, T5. The post-veraison water deficit has been reported to positively influence the red coloration and firmness of the berries [10]. Our results are similar to the ones reported by Faci et al. [36], which showed that only coloration was positively influenced by the irrigation deficit promoted by PI, while the hardness of the berries remained unaffected.

Irrigation reduction has been shown to promote berry coloration through the synthesis of bioactive compounds, the way that anthocyanins together with other phenolic compounds like flavonols can do in other table grape cultivars [12,17,36,37].

The combination of PI with biostimulation (B_PI_) significantly improved berry coloration, reducing the proportion of berries in Categories I and II, while increasing the ones in Categories III and IV to 76.0%, while T5_PI_ showed a 55.3% of its berries in commercial categories. Notwithstanding, not all the treatments responded in the same way to irrigation reduction. For example, T4, composed of AN extracts and *Bacillus*, improved the most in terms of berry color proportion in Categories III and IV; these biostimulants applied separately in T2 and T3 also promoted the commercial coloration by around 79%. Analogous to the irrigation reduction effect, flavonoid biosynthesis has been reported to be induced by AN extract [38]. In parallel, the plant growth-promoting rhizobacteria in grapevines are used to extend post-harvest life [39] by enhancing the synthesis of terpenes and phytohormones [40] or resveratrol [41]. On the other hand, other biostimulants like T1 did not, and similar to the untreated control T5, they maintained a similar berry color proportion under both irrigation programs.

Despite the seasonal variations in yield and quality parameters, which can contribute to the lack of significant results [36], the combined use of deficit irrigation applied during the post-veraison period and 2 years of biostimulation has increased the precocity and quality of table grapes, improving the maturity and coloration. The water reduction to which the vines were subjected lead them to accumulate a higher concentration of soluble solids and functional compounds that increased the skin coloration. Biostimulation has been proved to cause an increase in root density [29]. This increased root density promoted a greater soil water depletion and, consequently, a greater water stress in the plants under deficit irrigation, which then lowered their stem water potential from −0.82 to −0.89 MPa—although this effect was not significant with respect to the untreated plants [29].

Thus, the combination of both factors—biostimulation and deficit irrigation—caused an increase in the different quality parameters described above.

## 4. Materials and Methods

### 4.1. Experimental Conditions

The trial was carried out in a commercial orchard located in Alhama de Murcia, Region de Murcia, Spain (37°45′33″ N, 1°19′46″ W) during 2021 and 2022. The 3675 m^2^ experimental plot of table grapes (*Vitis vinifera* L.) cv. Sweet celebration, were at a planting frame of 3.5 m × 3.5 m in a clay soil (48% clay, 24% silt, 28% sand). The vines were covered by a net that prevent rainfall from percolating into the soil and were then irrigated through a drip irrigation system established by a line with 4 drippers per vine with a nominal flowrate of 3.8 L h^−1^. The crop evapotranspiration (ET_c_) was calculated according to the FAO method [42] by multiplying the crop coefficient (K_c_) with the reference evapotranspiration (ET_0_). Climate data were obtained from the agroclimatic station ‘AL-41′ located in La Calavera (Alhama de Murcia, Spain) belonging to the ‘Murcia Agrometeorological Information Service’ network [43].

For the study years, the period from June to August represented the peak of climatic demand, reaching the highest values for the reference crop evapotranspiration (ET_0_) of 7.4 mm and the vapor pressure deficit (VPD) of 2.1 to 2.8 kPa in 2021 and 2022, respectively. The region, characterized by a Mediterranean climate (Köppen “Bsh” classification [44]), experienced maximum temperatures of 35 °C and 36.2 °C during 2021 and 2022, respectively. Rainfall was significantly higher for the study period (May to November) in 2021 compared with 2022, totaling 186 and 96 mm (Table 1). More details on the experimental conditions have been described in [29].

### 4.2. Experimental Design and Treatments

A randomized trial design was established in plots corresponding to three adjacent rows of five vines, and the experimental unit was the three central vines located in the middle row, while the other two served as borders. In both years of study (2021 and 2022), five treatments were evaluated with four replicates (*n* = 4) based on the type of biostimulant applied and on a control without any application. The biostimulants were applied via fertigation at three phenological stages: (i) sprouting, at 61 and 40 days prior to flowering for 2021 and 2022, respectively; (ii) full bloom; and (iii) between the fruit set and pea-sized berries, at 48 and 22 days after full bloom (DAFB) in 2021 and 2022, respectively. The treatments corresponded to those described in Table 4.

Furthermore, to determine the effect of the use of biostimulants as agronomic management, their incorporation as a factor was analyzed at two levels: biostimulant application (T1–T4) vs. no application (T5) in both years of study. Additionally, in 2022, the plots were randomly divided into two sub-plots to evaluate irrigation scheduling as a factor at two levels: farmer irrigation (FI) and precision irrigation (PI). In the vines irrigated as FI, irrigation was scheduled according to the farmer’s traditional criteria, satisfying around 115% of the ET_c_. On the other hand, the deficit irrigation program, PI, was based on soil water depletion that was monitored with soil water status sensors. The irrigation time was reduced to maximize the soil water depletion, using an irrigation threshold of 10% of the field capacity. Differential irrigation for PI was applied during the post-veraison period, from 49 DAFB (5 July 2022) to 125 DAFB (19 September 2022). The rest of the season was under the FI program.

The composition of the biostimulant products is as follows: Amalgerol^®^ (Hechenbichler GmbH, Innsbruck, Austria) is formulated with seaweed and vegetable extracts, essential oils, distillate of paraffin oil, and 21% of total organic carbon. Seamac Rhizo^®^ (FMC Corporation, Philadelphia, PA, USA) is a combination of seaweed extract (*Ascophyllum nodosum*, 15%) with vegetable extracts, amino acids, and nutritional elements. Accudo^®^ (FMC Corporation, Philadelphia, PA, USA) is a plant growth-promoting rhizobacteria *Bacillus paralicheniformis* (RTI−184, 26 g L^−1^ [45]).

Pest and disease management were carried out in accordance with the commercial program equally for all the treatments. Among the treatments to all plots, it must be highlighted that the application of a foliar seaweed extract (*Ecklonia* spp.) was performed annually and that a mycorrhizal inoculant (*Glomus iranicum*, MycoUp 360^®^, Symborg, Murcia, Spain) was applied at fruit setting.

### 4.3. Field Measurements

#### 4.3.1. Crop Phenology

Crop phenology was monitored using the agrometeorological index of growing degree days (GDD) [46] according to Equation (1):

GDD = [(T_max_ + T_min_)/2)] − T_Base_, (1)

where T_max_ and T_min_ are the daily maximum and minimum air temperature, respectively, and T_Base_ is the base temperature for table grape growth, T_Base_ = 10 °C [47]. The GDD accumulation was calculated from sprouting (−62 Days After Full Bloom, DAFB).

#### 4.3.2. Soil and Plant Water Status

Soil water status was assessed in 2022 through an FDR probe model Drill & Drop (Sentek Technologies, Stepney, SA, Australia), which was installed in 3 replicates within each irrigation program in the wet bulb 10 cm apart from the dripper. These probes measure the soil volumetric water content, every 10 cm between 10 and 60 cm depth. Measures were obtained every minute and were averaged every 10 min. The data were registered in a datalogger and uploaded to *Irriman platform* [48]. The volumetric water content between a 20 and 40 cm depth was averaged and used to monitor the soil water status between blooming and veraison (pre-veraison), or veraison to the end of harvest (post-veraison). Whole-season raw data can be found at [29].

Plant water status was assessed through stem water potential at the solar midday (Ψ_s_) and was measured monthly from the start of the differential irrigation (49 DAFB) in 2022 using a pressure chamber type Scholander model Pump-Up (PMS Instrument Company, OR, USA) in 1 mature leaf per replicate, (*n* = 4 per treatment). The leaves were covered with foil bags 2 h prior to the measure.

### 4.4. Harvest

Fruit yield (t ha^−1^) was assessed in the 3 central vines of each plot. Harvest dates were based on marketable quality standard; therefore, they were distributed on 6 to 7 different dates, normally taking place between early June to late September (I: 6 August 2021, 29 July 2022; II: 10 August 2021, 16 August 2022; III: 17 August 2021, 22 August 2022; IV: 26 August 2021, 30 August 2022; V: 7 September 2021, 20 September 2022; VI: 20 September 2021, 30 September 2022; VII: 30 September 2021). All harvests were recorded to assess yield precocity. In addition to the yield data, the bunch number and bunch weight in the individual harvests were recorded.

### 4.5. Harvest Quality Traits

Harvest quality was assessed for the first harvest date for each year, respectively. The physical variables evaluated were the berry size and weight (*n* = 200 berries per replicate) and the hardness (*n* = 20 berries per replicate) using a digital caliper (Mitutoyo Co., Kawasaki, Japan), a precision scale (AX623 M-Pact, Sartorius, Germany), and a durometer (Hardmatic HH−300 Shore A, Mitutoyo, Kanagawa, Japan), respectively. The color proportion of the grapes was assessed based on a random sample of 200 berries per repetition, classifying the berries in 4 different color categories (Table 5). These correspond to (i) Category I: berries with green coloration and no commercial value; (ii) Category II: berries with reddish coloration; (iii) Category III: berries with 100% reddish coloration and optimal commercial condition; and, finally, (iv) Category IV: berries with very dark red coloration.

The quality traits analyzed in the grapes were the total soluble solid (TSS) and the titratable acidity (TA). They were measured through refractometry (ATAGO, Tokyo, Japan) and volumetric titration with NaOH to an end point of pH = 8.1 using an automatic titrator HI-84532 (Hanna Instruments Inc., Woonsocket, RI). Tartaric acid was considered the main acid present in the sample, and results were expressed as g L^−1^. The maturity index (MI) was calculated as the ratio between the TSS (°Brix) and the TA (%).

### 4.6. Statistical Analysis

The described variables were analyzed using linear mixed models (LMM) that included the effect of biostimulant treatments or factors and their interactions (biostimulant use, irrigation criterion, and year) in the fixed part of the model and the plots as the random part of the model (*p* < 0.05). In 2022, a hierarchical structure was added to the model, including sub-plots as random effects nested within the original plots. Prior to the analyses, assumptions were tested: the normality of the error distributions of each dependent variable was evaluated according to the Shapiro–Wilk test (*p* < 0.05), and the homoscedasticity of the variances was evaluated with the Levene test (*p* < 0.05), using absolute residuals to minimize the possible effect of outliers and improve the power of the test [49,50,51]. When significant differences by treatments or factors were detected, means were separated by Duncan’s test (*p* < 0.05). To evaluate the effect of treatments on berry color categories, contingency tables were elaborated for each category within each treatment, and then the chi-square test (*p* < 0.05) was used to determine if there was a significant association between color categories and treatments for each year and irrigation management method. Duncan’s test (*p* < 0.05) was used to compare the proportion of berries within the same category among treatments, while the Friedman test (*p* < 0.05) was used to evaluate the differences among color categories across the factors of biostimulation, irrigation management, and year. All statistical analyses were carried out using the InfoStat software version 2020e and its interface with R [52].

## 5. Conclusions

The combined use of deficit irrigation during the post-veraison period and two years of biostimulation has improved the precocity, maturity, and coloration of table grapes. This approach also mitigated alternate bearing, as observed in 2022. The highest yield was achieved with the combination of precision irrigation (PI) and T3 (biostimulant composed of microorganisms). The T4 treatment (seaweed extracts and microorganisms) maintained a yield above 40 t ha^−^¹ under both irrigation programs.

The accumulated effect of two years of biostimulation improved the physical quality of grapes under FI, with the most significant size increase observed in T2 (seaweed extract). Grape coloration and chemical quality traits (TSS and MI) were enhanced when combining biostimulants with PI, especially in T4.

On average, this management strategy, which combines biostimulation and irrigation reduction, did not negatively affect production. In fact, it reduced irrigation costs, contributing to the sustainability of the crop. Additionally, the early market entry of these high-quality grapes allows farmers to capitalize on higher prices, further boosting their revenue. This approach supports efficient and sustainable farming practices, showing high potential for improving grape yield and quality in drought-prone regions.

## Figures and Tables

**Table 1:** Seasonal rainfall, evapotranspiration (ET_0_), and irrigation water applied for the season 2021 and 2022 divided into pre-veraison (Pre) and post-veraison (Post) periods. Increase in the 20 to 40 cm depth soil water content (θ) and average stem water potential (Ψ_s_) for farmer and precision irrigation programs in 2022.

Year	Irrigation Program	Rainfall (mm)			ET_0_ (mm Day^−1^)			Irrigation (m^3^ ha^−1^)			Δθ_20–40cm_ (%)		Ψ_s_ (MPa)
		Pre	Post	Total	Pre	Post	Max	Pre	Post	Total	Pre	Post	Post
2021	Farmer	110	76	186	5.77	4.73	7.35	1218	3193	4411			
2022	Farmer	39	57	96	6.35	4.66	7.43	1675	2728	4403	9.90	2.06	−0.66
2022	Precision							1454	1613	3067		−0.03	−0.86

Each season was considered to run from May to November. Veraison took place at 1000 °C GDD, corresponding to 11 July 2021 and 9 July 2022, respectively. The soil water content period for post-veraison was monitored until the irrigation recovery on 19 September 2022.

**Table 2:** Physical quality traits (berry size, weight, and hardness) of the fruits from vines subjected to different biostimulation treatments during the 2021 and 2022 season under two irrigation programs.

Year (Y)	Irrigation	Biostimulation	Treatment	Berry Size		Berry Weight		Hardness	
	(I)	(B)	(T)	mm		g		Shore A Scale	
2021	Farmer	Yes	T1	20.38		6.38		54.95	
			T2	20.49		6.54		51.12	
			T3	20.27		6.29		54.33	
			T4	20.36		6.48		57.65	
		No	T5	20.26		6.33		50.00	
			*ANOVA*	*0.9897 ^ns^*		*0.9347 ^ns^*		*0.4425 ^ns^*	
2022	Farmer	Yes	T1	18.76	b	5.27	a	61.29	b
			T2	19.40	a	5.00	a	65.37	b
			T3	18.66	bc	5.22	a	77.63	a
			T4	18.39	c	4.98	a	60.64	b
		No	T5	16.86	d	3.97	b	62.15	b
			*ANOVA*	<0.0001 ***		<0.0001 ***		<0.0001 ***	
	Precision	Yes	T1	18.20		4.81		62.61	
			T2	17.94		5.01		65.78	
			T3	18.40		4.98		70.37	
			T4	18.70		5.03		63.11	
		No	T5	18.29		4.70		68.08	
			*ANOVA*	*0.0506 ^ns^*		*0.1124 ^ns^*		*0.2208 ^ns^*	
	Farmer								
			Y	<0.0001 ***		<0.0001 ***		<0.0001 ***	
			T	<0.0001 ***		0.0399 *		<0.0001 ***	
			Y × T	<0.0001 ***		0.0295 *		<0.0001 ***	
2022									
			B	*<* 0.0001 ***		<0.0001 ***		*0.6237 ^ns^*	
			I	0.0479 *		*0.8127 ^ns^*		*0.6313 ^ns^*	
			B × I	*<* 0.0001 ***		0.0001 ***		0.0257 *	

Means, *n* = 4. Different letters for the same parameter indicate significant differences between treatments under the same condition according to Duncan’s test. (*p* < 0.05). The factors and their interaction were analyzed considering (i) the individual treatments (T) and annual (Y) effects under the farmer irrigation program and (ii) the biostimulation (B) and irrigation (I) effects in 2022. *: *p* < 0.05; **: *p* < 0.01; ***: *p* < 0.001; *^ns^*: not significant.

**Table 3:** Chemical quality traits, total soluble solid (TSS), titratable acidity (TA), and maturity index (MI) for the different treatments in season 2021 and 2022 under two irrigation programs.

Year (Y)	Irrigation	Biostimulation	Treatment	TSS		TA		MI	
	(I)	(B)	(T)	Brix		g L^−1^			
2021	Farmer	Yes	T1	18.35		5.28		34.75	
			T2	18.42		5.65		32.60	
			T3	18.69		6.05		30.89	
			T4	18.54		5.18		35.79	
		No	T5	18.24		6.15		29.66	
			*ANOVA*	*0.7091 ^ns^*		*0.5084 ^ns^*		*0.6427 ^ns^*	
2022	Farmer	Yes	T1	16.80		3.80		44.21	
			T2	17.65		3.80		46.45	
			T3	16.25		3.80		42.76	
			T4	16.75		3.80		44.08	
		No	T5	16.25		3.75		43.33	
			*ANOVA*	*0.2876 ^ns^*		*0.0723 ^ns^*		*0.2621 ^ns^*	
	Precision	Yes	T1	17.60	a	3.75	a	46.93	a
			T2	17.45	a	3.75	a	46.53	a
			T3	17.35	a	3.60	b	48.19	a
			T4	17.70	a	3.80	a	46.58	a
		No	T5	16.55	b	3.75	a	44.13	b
			*ANOVA*	0.0125 *		0.0009 ***		0.0048 **	
	Farmer								
			*Y*	<0.0001 ***		<0.0001 ***		<0.0001 ***	
			*T*	*0.3551 ^ns^*		*0.6807 ^ns^*		*0.6308 ^ns^*	
			*Y × T*	*0.7941 ^ns^*		*0.6278 ^ns^*		*0.7754 ^ns^*	
2022									
			*B*	0.0133 *		*0.6408 ^ns^*		0.0154 *	
			*I*	0.0205 *		0.0088 **		0.0008 ***	
			*B × I*	*0.5495 ^ns^*		*0.1686 ^ns^*		*0.2433 ^ns^*	

Means, *n* = 4. Different letters for the same parameter indicate significant differences between treatments under the same condition according to Duncan’s test. (*p* < 0.05). The factors and their interaction were analyzed considering (i) the individual treatments (T) and annual (Y) effects under the farmer irrigation program and (ii) the biostimulation (B) and irrigation (I) effects in 2022. *: *p* < 0.05; **: *p* < 0.01; ***: *p* < 0.001; ^ns^: not significant.

**Table 4:** Biostimulation treatments applied via fertigation at different phenological stages of the vine during 2021 and 2022.

Treatment	Sprouting	Full Bloom	Fruit Set to Pea-Sized Berries
	L ha^−1^		
T1 ^A^: Amalgerol^®^	10	5	5
T2 ^A^: Seamac Rhizo^®^	5	5	5
T3 ^A^: Accudo^®^	1	1	1
T4: Seamac Rhizo^®^ +Accudo^®^	4 1	4 1	4 1
T5: Control	-	-	-

^A^ During the pre-conditioning year (2021), treatments T1, T2, and T3 were applied together with another seaweed extract biostimulant, Seamac PCT^®^ (*Ascophyllum nodosum*, 15%), via foliar spray at 0.30% at 44 days before bloom and at 12 and 20 days after full bloom.

**Table 5:** Berry color categories used in the trial.

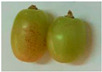	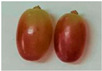	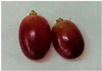	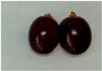
Category I	Category II	Category III	Category IV

## Data Availability

Data will be made available on request.
